# Effectiveness of integrated management on hypertension and mortality in rural China: A CHHRS study

**DOI:** 10.1016/j.isci.2024.110865

**Published:** 2024-08-31

**Authors:** Chao Yu, Yumeng Shi, Peixu Zhao, Tao Wang, Lingjuan Zhu, Wei Zhou, Huihui Bao, Xiaoshu Cheng

**Affiliations:** 1 Department of Cardiovascular Medicine, the Second Affiliated Hospital, Jiangxi Medical College, Nanchang University, Nanchang of Jiangxi, China; 2 Center for Prevention and Treatment of Cardiovascular Diseases, the Second Affiliated Hospital, Jiangxi Medical College, Nanchang University, Nanchang of Jiangxi, China; 3 Jiangxi Provincial Cardiovascular Disease Clinical Medical Research Center, Nanchang of Jiangxi, China; 4 Jiangxi Sub-center of National Clinical Research Center for Cardiovascular Diseases, Nanchang, Jiangxi, China

**Keywords:** Medicine, Internal medicine, Public health

## Abstract

A study was conducted to investigate whether an integrated management (IM) model led by public healthcare providers is effective in reducing cardiovascular disease (CVD)-specific and all-cause mortality rates in low-income rural populations with hypertension. The study recruited 14,234 patients with hypertension aged 18 years or older and allocated them to either an IM group or a usual care (UC) group. During a median follow-up of 48.0 months, the incidences of CVD-specific and all-cause deaths were lower in the IM group than in the UC group. The hazard ratios for CVD-specific mortality and all-cause mortality among patients in the IM group were 0.60 and 0.62, respectively. The results showed that the IM model led by public health providers resulted in clinically significant reductions in CVD-specific and all-cause mortality rates in low-income rural populations with hypertension.

## Highlights

• Integrated management is central to rural healthcare systems
• Comprehensive hypertension management impacts patient
• This study offers valuable reference for hypertension management

## Graphical abstract

**Figure:**
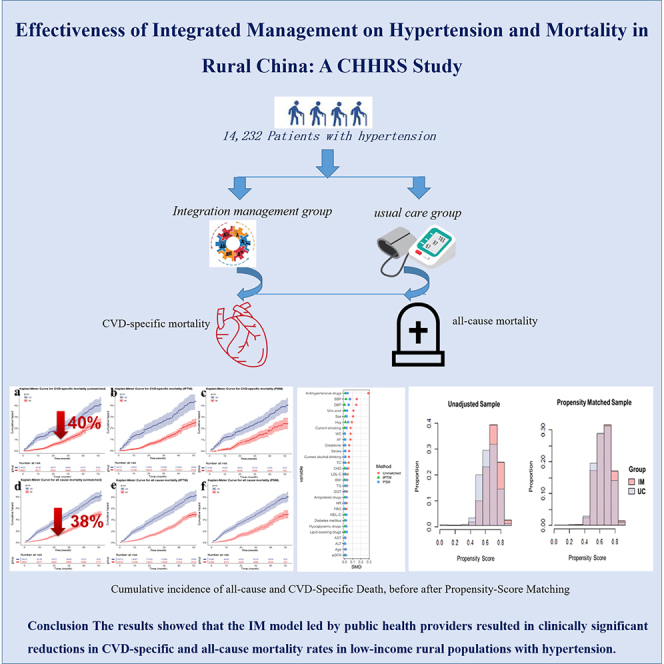

## Introduction

Hypertension is a significant global public health concern that can lead to cardiovascular and cerebrovascular diseases, such as stroke, coronary heart disease (CHD), and heart failure, as well as mortality.^1^^,^^2^ Between 1990 and 2019, the global prevalence of high blood pressure among individuals aged 30–79 years doubled from an estimated 650 million to approximately 1.28 billion.^3^ Prevention and treatment of hypertension is only provided in primary and community healthcare institutions, and the progression of hypertension and related complications can be effectively controlled through the use of more cost-efficient antihypertensive drugs.^1^^,^^4^^,^^5^ However, a global disease burden study revealed a shift in the distribution of hypertension prevalence from high-income region to low- and middle-income region, with an increasing trend observed in many of these region such as central and eastern Europe, central Asia, Oceania, and southern Africa.^3^^,^^6^ Poor control rate of hypertension among low-income individuals may stem from challenges in accessing high-quality healthcare services, limited availability and affordability of antihypertensive medications, insufficient health literacy, and suboptimal adherence to prescribed treatments.^7^^,^^8^^,^^9^ In areas with limited medical resources, non-physical community healthcare providers have demonstrated effective intervention measures to overcome obstacles, improve hypertension control rates, and reduce the risk of cardiovascular disease (CVD)-specific death in low-resource environments.^10^^,^^11^^,^^12^

The management of hypertension in both rural and urban areas necessitates collaboration and coordination among patients, medical personnel, and healthcare systems. However, owing to limited resources in rural regions, there are often impediments at all levels mentioned previously.^9^^,^^13^^,^^14^ Therefore, we must leverage the power of public healthcare providers to establish an integrated management (IM) model for patients with hypertension in rural areas. This will enable us to harmonize management strategies across health systems, medical personnel, public healthcare providers, and patients. No study has assessed the effect of IM on CVD-specific and all-cause mortality among low-income individuals residing in rural areas.

The implementation of IM strategies for individuals with hypertension in low-income areas, particularly rural regions, will have a significant impact on public health outcomes. Therefore, we utilized the population from the China H-type Hypertension Registration Study (CHHRS) to validate the effectiveness of non-physician-led comprehensive management and conventional treatment in reducing CVD-specific and all-cause mortality among patients with hypertension.

## Results

### Characteristics of the cohort

Overall, 14,232 patients with hypertension (mean age 63.8 years; 47.2% male participants) were included in this study. Over a median follow-up of 48.0 months, the incidences of CVD-specific and all-cause deaths were 2.29% and 4.69% in the IM group, and 3.83% and 7.68% in the usual care (UC) group, respectively. Table 1 displays the baseline characteristics of the IM and conventional care groups across the original, inverse probability weighting (IPTW), and propensity score (PS)-matched datasets. Compared with those in the routine nursing care group, we observed that males in the IM group exhibited larger waist circumference (WC), higher rates of current smoking and drinking, and elevated systolic and diastolic blood pressure (DBP) values. Patients with a history of stroke had a higher utilization rate of antihypertensive drugs than those with CHD or atrial fibrillation (AF). Additionally, patients in the former group displayed increased levels of homocysteine (Hcy), total cholesterol (TC), and low-density lipoprotein cholesterol (LDL-C), while exhibiting decreased levels of uric acid and creatinine. In the PS-matched dataset, we observed that differences between the IM and conventional care groups were only evident in systolic blood pressure (SBP), DBP, and Hcy levels; however, these values were closely aligned with one another and lacked clinical significance. There was no statistical difference in social demographic characteristics between the two groups (see Table S2). The IPTW dataset revealed no significant differences in baseline characteristics between the two groups.

**Table 1:** Characteristics of patients receiving integration management or usual care, before and after propensity-score matching

Characteristic	Unmatched patients				IPTW-matched patients				PS-matched patients			
	UC	IM	*p* value	SMD	UC	IM	*p* value	SMD	UC	IM	*p* value	SMD
Participants	4625	9607			14271.7	14231.8			4586	4586		
Males, *n* (%)	2022(43.7)	4698(48.9)	<0.001	0.104	6644.4(46.6)	6695.6(47.0)	0.600	0.010	2006(43.7)	2062(45.0)	0.248	0.025
Age, year, mean (SD)	63.84(10.11)	63.80(8.98)	0.806	0.004	63.95(9.94)	63.83(9.01)	0.495	0.013	63.84(10.09)	63.97(9.07)	0.514	0.014
BMI,kg/m^2^, mean (SD)	23.52(3.60)	23.65(3.80)	0.052	0.035	23.63(3.57)	23.63(4.18)	0.966	0.001	23.53(3.60)	23.47(3.53)	0.428	0.017
WC, cm, mean (SD)	83.35(9.80)	84.07(9.86)	<0.001	0.073	83.86(9.75)	83.86(9.88)	0.993	<0.001	83.36(9.79)	83.39(9.67)	0.909	0.002
Current smoking, *n* (%)	1089(23.5)	2573(26.8)	<0.001	0.075	3652.3(25.6)	3656.2(25.7)	0.904	0.002	1081(23.6)	1126(24.6)	0.282	0.023
Current alcohol drinking, *n* (%)	927(20.0)	2140(22.3)	0.003	0.055	3026.9(21.2)	3054.9(21.5)	0.740	0.006	924(20.1)	940(20.5)	0.697	0.009
SBP, mmHg, mean (SD)	146.60(17.01)	149.26(18.20)	<0.001	0.151	148.77(18.07)	148.46(17.95)	0.385	0.017	146.66(16.96)	147.80(17.55)	0.002	0.066
DBP, mmHg, mean (SD)	87.89(10.29)	89.42(10.93)	<0.001	0.144	88.92(10.47)	88.92(10.88)	0.991	<0.001	87.92(10.27)	88.46(10.88)	0.015	0.051
Heart rate, beats/min, mean (SD)	76.91(14.19)	76.55(14.17)	0.157	0.025	76.81(14.20)	76.69(14.34)	0.648	0.009	76.82(14.11)	76.73(14.17)	0.762	0.006

**Past diagnoses, *n* (%)**												

Diabetes mellitus^a^	828(17.9)	1790(18.6)	0.303	0.019	2663.6(18.7)	2641.5(18.6)	0.889	0.003	820(17.9)	796(17.4)	0.528	0.014
Stroke	275(5.9)	707(7.4)	0.002	0.057	1012.4(7.1)	988.1(6.9)	0.763	0.006	274(6.0)	284(6.2)	0.694	0.009
CHD	268(5.8)	461(4.8)	0.013	0.044	716.5(5.0)	721.4(5.1)	0.902	0.002	263(5.7)	242(5.3)	0.360	0.020
AF	164(3.5)	224(2.3)	<0.001	0.072	390.2(2.7)	390.5(2.7)	0.974	0.001	155(3.4)	148(3.2)	0.726	0.009

**Medications at baselines, *n* (%)**												

Antihypertensive drugs	2562(55.4)	6666(69.4)	<0.001	0.292	9319.4(65.3)	9229.0(64.8)	0.600	0.009	2542(55.4)	2624(57.2)	0.088	0.036
Hypoglycemic drugs	256(5.5)	499(5.2)	0.418	0.015	753.1(5.3)	769.2(5.4)	0.767	0.006	253(5.5)	232(5.1)	0.351	0.020
Lipid-lowering drugs	172(3.7)	334(3.5)	0.495	0.013	515.0(3.6)	507.9(3.6)	0.906	0.002	170(3.7)	149(3.2)	0.254	0.025
Antiplatelet drugs	194(4.2)	352(3.7)	0.134	0.027	558.9(3.9)	547.5(3.8)	0.844	0.004	191(4.2)	177(3.9)	0.489	0.016

**Initial laboratory tests, mean (SD)**												

Hcy, μmol/L	17.30(10.12)	18.28(11.57)	<0.001	0.090	18.11(12.23)	17.98(11.05)	0.625	0.011	17.26(10.09)	17.71(10.69)	0.039	0.043
FBG, mmol/L	6.16(1.60)	6.20(1.61)	0.187	0.024	6.20(1.63)	6.19(1.60)	0.841	0.004	6.16(1.60)	6.16(1.62)	0.999	<0.001
TC, mmol/L	5.12(1.11)	5.18(1.12)	0.005	0.050	5.17(1.12)	5.16(1.12)	0.591	0.010	5.13(1.11)	5.13(1.12)	0.967	0.001
TG, mmol/L	1.78(1.22)	1.82(1.29)	0.077	0.032	1.82(1.27)	1.81(1.29)	0.837	0.004	1.78(1.21)	1.80(1.31)	0.520	0.013
AST, mmol/L	26.91(21.46)	26.69(12.18)	0.426	0.013	26.78(17.04)	26.73(12.77)	0.840	0.004	26.71(14.11)	26.77(13.58)	0.819	0.005
ALT, mmol/L	20.40(20.70)	20.55(14.23)	0.630	0.008	20.51(17.74)	20.48(14.62)	0.920	0.002	20.24(15.40)	20.34(15.13)	0.747	0.007
Uric acid, mmol/L	409.92(121.67)	423.17(119.81)	<0.001	0.110	418.81(123.56)	419.10(118.88)	0.901	0.002	409.82(121.53)	413.35(118.41)	0.159	0.029
Creatinine, μmol/L	74.82(65.59)	71.84(32.53)	<0.001	0.057	72.98(45.90)	73.05(45.50)	0.952	0.002	71.43(38.69)	71.68(37.19)	0.753	0.007
GGT, U/L	32.38(42.50)	33.64(43.21)	0.100	0.029	33.24(42.77)	33.24(42.93)	0.997	<0.001	32.38(42.58)	32.75(43.97)	0.680	0.009
HDL-C, mmol/L	1.57(0.41)	1.57(0.43)	0.206	0.023	1.57(0.41)	1.57(0.44)	0.782	0.005	1.58(0.41)	1.57(0.44)	0.355	0.019
LDL-C, mmol/L	2.96(0.81)	2.99(0.82)	0.033	0.038	2.99(0.82)	2.98(0.82)	0.549	0.011	2.97(0.81)	2.97(0.81)	0.900	0.003
eGFR, mL/min/1.73 m^2^	88.17(21.32)	88.18(19.67)	0.974	0.001	87.91(20.67)	88.06(20.13)	0.698	0.008	88.59(20.56)	88.27(19.93)	0.459	0.015

Abbreviation: UC, usual care; IM, integration management; BMI, body mass index; WC, waist circumference; DBP, diastolic blood pressure; GGT, γ-glutamyltransferase; TC, total cholesterol; TG, triglycerides; HDL-C, high-density lipoprotein cholesterol; LDL-C, low-density lipoprotein cholesterol; Hcy, homocysteine; eGFR, estimated glomerular filtration rate.

a Diabetes mellitus was defined as self-reported physician diagnosis of diabetes or FBG concentration ≥7.0 mmol/L or use of glucose-lowering drugs.

Figure S3 illustrates the standardized mean difference between the two sets of baseline variables across the three datasets, indicating that the baseline data of the PS-matched dataset and IPTW dataset of the integration management group were well balanced with those of the UC group. The distribution of the estimated propensity scores for receiving integration management among patients who received UC is shown in Figures S4 and S5. The c-statistic for the PS model as the dependent variable was 0.85, all the aforementioned data indicating good prediction. The hazard ratios (HRs; with 95% confidence intervals [CIs]) for the composite endpoint for all variables included as covariates in the Cox multivariate model, with inverse probability weighting by propensity score, are shown in Table S4.

### Study endpoints

**Figure 1:**
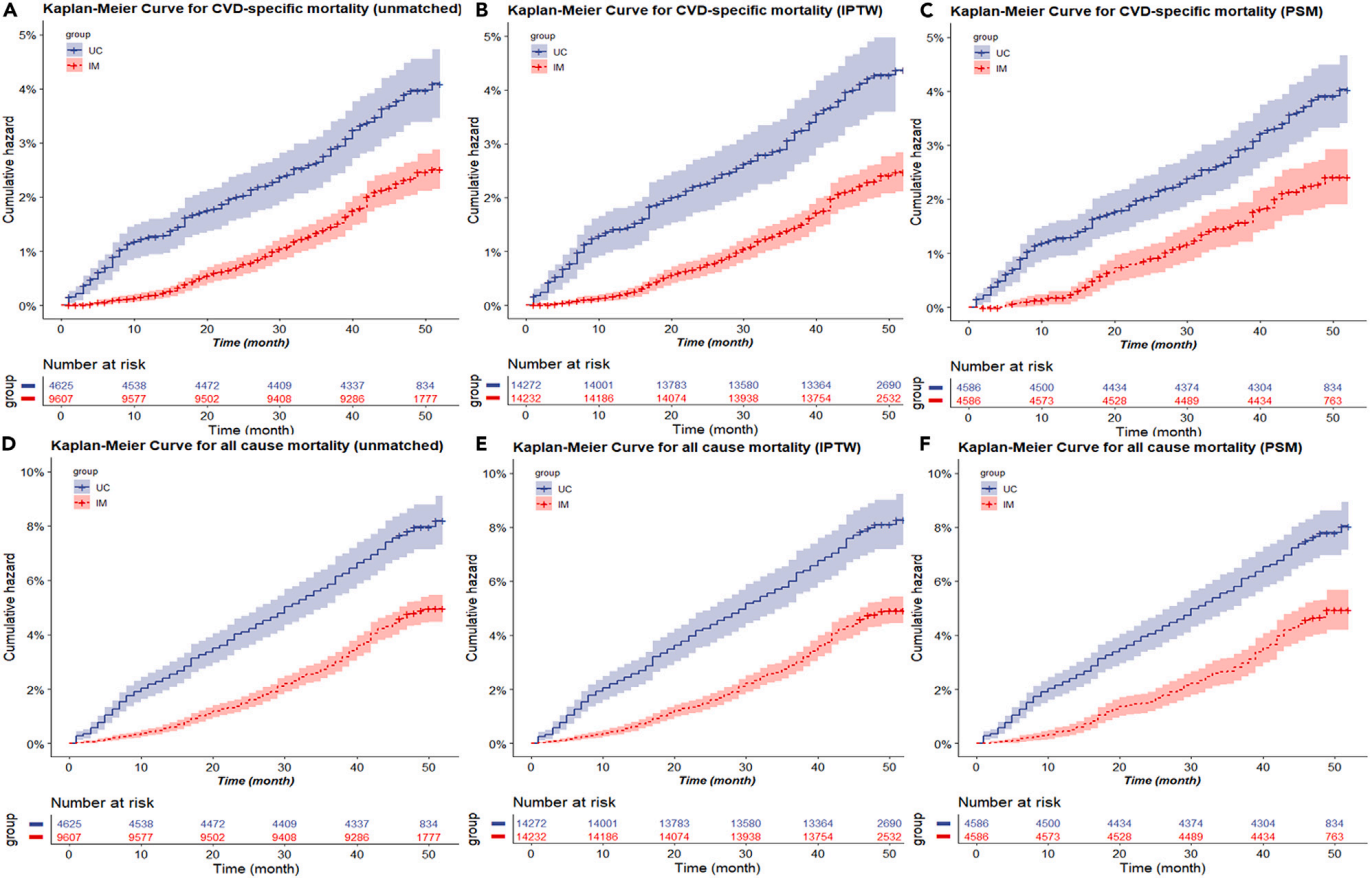
Cumulative incidence of CVD-specific and all-cause mortality, before after propensity-score matching (A) Kaplan-Meier survival curve for CVD-specific mortality (unmatched). (B) Kaplan-Meier survival curve for CVD-specific mortality (IPTW). (C) Kaplan-Meier survival curve for CVD-specific mortality (PSM). (D) Kaplan-Meier survival curve for all-cause mortality (unmatched). (E) Kaplan-Meier survival curve for all-cause mortality (IPTW). (F) Kaplan-Meier survival curve for all-cause mortality (PSM). The shaded areas represent pointwise 95% confidence intervals.

We observed a statistically significant 40% and 38% reductions in the risks of CVD-specific (HR: 0.60, 95% CI: 0.49, 0.73) and all-cause mortality (HR: 0.62, 95% CI: 0.54, 0.72) among patients in the IM group compared with those in the control group, as demonstrated by adjusted model. Additional Cox proportional hazards regression analyses yielded similar results (Table 2) in both the IPTW and PS-matched datasets (both unadjusted and adjusted models), to assess the impact on CVD-specific and all-cause mortality within each group. The findings indicated that the IM model group exhibited significant advantages in terms of CVD-specific and all-cause mortality. The Kaplan-Meier survival curve results demonstrated that patients in the IM group had a lower incidence of cumulative CVD-specific and all-cause mortality, compared with those in the UC group across mismatched datasets (Figures 1A and 1D), IPTW dataset (Figures 1B and 1E), and PS matched dataset (Figures 1C and 1F). The stacked cumulative incidence function (CIF) curve shows the similar result (Figure S7).

**Table 2:** Effectiveness of a public health-care provider-led integration management on the CVD-specific and all-cause mortality in the multivariable analysis, and propensity-score analyses

Methods	H*R* (95%CI), *p* value
**CVD-specific mortality**	

Multivariable analysis^a^	0.60 (0.49, 0.73) <0.0001
Propensity-score analyses	
With inverse probability weighting^b^	0.53 (0.43, 0.66) <0.0001
With matching^c^	0.58 (0.46, 0.75) <0.0001
Adjusted for propensity score^d^	0.57 (0.45, 0.73) <0.0001

**All-cause mortality**	

Multivariable analysis^a^	0.62 (0.54, 0.72) <0.0001
Propensity-score analyses	
With inverse probability weighting^b^	0.59 (0.51, 0.68) <0.0001
With matching^c^	0.60 (0.50, 0.71) <0.0001
Adjusted for propensity score^d^	0.59 (0.50, 0.71) <0.0001

Crude analysis was adjusted for none.

Abbreviations: HR, hazard ratio; CI, confidence interval.

a Shown is the hazard ratio from the multivariable Cox proportional-hazards model, with additional adjustment for age, sex, BMI, WC, current smoking, current alcohol drinking, SBP, DBP, HR, diabetes mellitus, stroke, CHD, AF, HCY, TCHO, TG, LDL-C, HDL-C, UA, eGFR, AST, ALT, GGT, creatinine, hypoglycemic drugs, lipid-lowering drugs, antiplatelet drugs, antihypertensive drugs, and laboratory tests on presentation. The analysis included all 14,232 patients.

b Shown is the primary analysis with a hazard ratio from the multivariable Cox proportional-hazards model with the same covariates with inverse probability weighting according to the propensity score. The analysis included 28,504 patients (14,232 who received integration management and 14,272 who did not).

c Shown is the hazard ratio from a multivariable Cox proportional-hazards model with the same covariates with matching according to the propensity score. The analysis included 9,172 patients (4,586 who received integration management and 4,586 who did not).

d Shown is the hazard ratio from a multivariable Cox proportional-hazards model with the same strata and covariates, with additional adjustment for the propensity score. The analysis included 9,172 patients (4,586 who received integration management and 4,586 who did not).

### Sensitivity analyses

Further internal validation for randomly selected data from 10 out of 17 townships for the same analysis showed similar reductions in the risks of CVD-specific and all-cause mortality among patients in the IM group compared with those in the control group (see Table S3). The findings were similar when the main analyses were conducted using competing risk regressions. In addition, the results of analyses that perform multiple imputations for covariates or excluded participants who died within the first six months of follow-up were still consistent with those of the primary analysis (see Table S3).

## Discussion

This large-scale prospective cohort study demonstrated that an IM model led by public health providers significantly reduced the risk of CVD-specific and all-cause mortality in rural patients with hypertension. Furthermore, this effect was consistent across the different datasets.

The results of this study hold immense importance for global public health. In low- and middle-income countries and regions, the increasing prevalence of hypertension coupled with inadequate comprehensive management of patients with hypertension remains a leading cause of cardiovascular-related mortality in these areas.^15^^,^^16^ The IM model examined in our study comprised several components. Patients who voluntarily opted for government-issued antihypertensive medications under welfare schemes were included in the IM program. Public health providers recorded patient-specific information and provided services such as blood pressure monitoring, health education, and regular follow-ups (once every two months). Subsequently, township medical staff trained on standardized prescription issued prescriptions to patients, who then obtained the corresponding medications from public health providers. Adherence can be enhanced by improving patients’ access to affordable medications and providing them with comprehensive health education.^7^^,^^17^

A systematic evaluation conducted in numerous high-income regions demonstrated that the implementation of IM strategies has a favorable impact on the prevention and treatment of patients with hypertension.^12^ In contrast to our study, these studies primarily targeted urban populace.^18^^,^^19^^,^^20^ A multifaceted strategy that has been proven to be effective should be promoted in rural China to reduce the incidence and mortality of hypertension-related diseases. Meanwhile, the widespread use of interventions by healthcare centers requires changes in health policies. Our findings suggest that it is imperative for relevant provincial medical specialists to provide regular standardized prescription training to county- and township-level medical staff, in order to effectively manage severe CVDs and prevent deaths caused by primary hypertension and further development of related conditions. Additionally, we emphasized the importance of a close collaboration between public health providers and hospital staff.

The previous research shows that the rural Chinese death reporting system lacks reliability because most deaths occur in unsupervised home settings without medical oversight.^21^^,^^22^ Furthermore, the majority of patients died after discharge from the hospital; thus, access to their medical records via the death registration system was precluded.^23^ In this study, we obtained the medical records of the majority of patients who died in the hospital through their physicians’ community healthcare provider office, township hospital, county, or city hospital. For those who died outside of a medical facility, we acquired their medical history, previous medical records, data on CVD-related symptoms and signs, as well as death certificates from either close relatives or attending medical staff. The Endpoint Committee was responsible for determining the cause of death.

The IM presented in this study encompasses the core of rural healthcare systems. A systematic and comprehensive approach to hypertension management at the patient, public health provider, and medical staff levels holds significant implications for public health. Furthermore, this real-world observational study can serve as a valuable reference for the clinical management of patients with hypertension in other rural areas of China.

### Limitations of the study

The following limitations should be considered when interpreting the results. Observational studies inherently have immeasurable confounding factors; however, we utilized inverse probability weighting and PS matching to improve the equalization between groups and minimize the potential effects of confounders on exposure factors and death events, to achieve “minic-randomization” effect. Despite this extensive adjustment, it is still possible that some amount of unmeasured confounding remains. Furthermore, our study lacks data on the correlation between patient financial status and health insurance costs, which has significant implications for further advancement of our findings. Finally, the impact of IM may have been underestimated due to potential behavioral changes in patients who received conventional care due to the measures implemented by the researchers for outcome evaluation.

In this study it was discovered that compared with the UC group, the risk of CVD-specific and all-cause mortality in the IM group led by public health providers was significantly reduced in the rural regions. Future randomized clinical trials are necessary to further validate the findings of this study and develop practical public health policies for promoting CVD-specific and all-cause mortality rates reduction among patients with hypertension in low- and middle-income rural areas.

## Resource availability

### Lead contact

Further information and requests for resources and data should be directed to the lead contact, Dr. Xiaoshu Cheng (xiaoshumenfan126@163.com).

### Materials availability

This study did not generate new data.

### Data and code availability

• Data analyzed can be made available to others upon reasonable requests to the lead contact, Dr. Xiaoshu Cheng (xiaoshumenfan126@163.com), if access is granted. A proposal with a detailed description of the study objectives and the statistical analysis plan will be needed for evaluation of the reasonability of requests.
• The code related to the attribution model can be accessed by reaching out to the lead contact.

## Acknowledgments

Thanks to all the investigators and subjects who participated in the CHHRS. This work was supported by the Cultivation of backup projects for National Natural Science Foundation of China (82460670), Cultivation of Backup Projects for National Science and Technology Awards of China (20223AEI91007), Jiangxi Science and Technology Innovation Base Plan - Jiangxi Clinical Medical Research Center(20223BCG74012), 10.13039/501100004479Jiangxi Provincial Natural Science Foundation, China (20224BAB206090 and 20232BAB206140), Jiangxi Provincial Health Commission Science and Technology Project (202130440, 202210495, and 202310528), Jiangxi Provincial Drug Administration Science and Technology Project (2023JS26), and Fund project of the Second Affiliated Hospital of Nanchang University (2021efyA01 and 2023efyA05).

## Author contributions

C.Y.: study concept and design, acquisition of data, data analysis, data interpretation, drafting of the manuscript. Y.S.: revision of the manuscript for important intellectual content. P.Z., T.W., L.Z., and T.W.: acquisition of data, critical review and revision of the manuscript for important intellectual content. H.B. and X.C.: study concept and design, acquisition of data, data management, critical review and revision of the manuscript for important intellectual content.

## Declaration of interests

The authors declare no competing interests.

## STAR★Methods

### Key resources table

**Table:** 

REAGENT or RESOURCE	SOURCE	IDENTIFIER
**Software and algorithms**		

R version	The R Project for Statistical Computing	http://www.r-project.org

### Experimental model and study participant details

This study were approved by the ethics committee of the Institute of Biomedicine, Anhui Medical University (NO. CH1059) and the Second Affiliated Hospital of Nanchang University (NO. 2018019). Informed consent was obtained from all subjects and/or their legal guardian(s). The database consulted derives from the China H-type Hypertension Registry Study (registration number: ChiCTR1800017274; 20/07/2018).

#### Study design and participants

The study population was derived from the hypertension registration study which served as a prospective observational study. This study aimed to establish a population-based H-type hypertension registry cohort and explore the prevalence, treatment status, and related factors that influence the prognosis of H-type hypertension. The eligibility criteria for the present study were as follows: participants aged 18 years or older; patients diagnosed with hypertension meeting the following specific criteria: SBP ≥140 mmHg and/or DBP ≥90 mmHg, or use of antihypertensive medication orally within two weeks; provision of informed consent. The exclusion criteria comprised patients with psychiatric disorders or impaired communication abilities, as well as short-term residents and those unable to comply with the required follow-up protocol. The study was approved by the Ethics Committees of the Institute.

We enrolled 14,234 patients with hypertension from 17 villages and towns in Wuyuan County at baseline in 2018 and allocated them into two groups: IM group (*n* = 9607) and routine treatment group (*n* = 4625). After an average follow-up period of 48 months, the final analysis included 9,607 patients in the IM group, and all but two patients were followed up in the conventional care group, resulting in a total of 4,625 patients (see Figure S1).

### Method details

This study included patients with hypertension who voluntarily elected to receive government-introduced medications and standardized prescription that benefited the public. Patients who agreed to take the standardized prescription were automatically enrolled in the IM group, whereas those who declined were placed in the routine care group. The integration management group primarily comprised the following four components: 1) standardized prescription (see Table S1) training for township medical staff, 2) Patients undergoing treatment with Huimin medication, 3) patients monitored at bi-monthly intervals by healthcare professionals employed in the public sector and 4) a regular health education plan. The routine care groups comprised preexisting primary medical services, with the exception of standardized training on hypertension and antihypertensive treatment for township medical staff. They received no further instructions and relied primarily on ordinary village doctors for their UC (see Figure S2). Demographic information, including sex, age, tobacco and alcohol use, drug use history, and medical records was obtained through face-to-face questionnaires at baseline. Medical examination was conducted by professional physicians, encompassing measurements of height, weight, blood pressure, and other vital signs. Venous blood samples were collected from patients who had undergone an overnight fast of 8–12 h before 7:00 a.m. each morning, followed by laboratory analysis of blood biochemical and liver and kidney function parameters. Body mass index (BMI) was calculated as body weight in kilograms per square of height in meters (kg/m^2^). The eGFR was calculated using the Chronic Kidney Disease Epidemiology Collaboration (CKD-EPI) equation.^24^ Among them, the disease and medication history obtained from the questionnaire are cross-validated with medical records provided by the medical insurance bureau. The drug utilization data of hospitalized patients was extracted from medical records, while for non-hospitalized patients, it is derived from outpatient records and patient self-reports.

#### Outcomes

The primary endpoint was CVD-specific mortality, and the secondary endpoint was all-cause mortality. Participants were followed up from their study entry date in 2018 until death or the end of follow-up (August 15, 2022), whichever came first. Vital status was ascertained through linkage to the Chinese Centers for Disease Control and Prevention (CDC) in Jiangxi Province and the national health insurance system (which was linked with hospitalization records), and then cross-checked with the death registration database of the Public Security Bureau, as well as through follow-up visits (e.g., telephone interviews, consultation with village doctors, and door-to-door interviews). The cause of death was further determined by an independent Endpoint Adjudication Committee based on the last hospital’s medical records. The International Classification of Diseases, Tenth Revision (ICD-10) codes were used to classify deaths resulting from CVD, including heart disease (I00-109, I11, I13, I20-151) or cerebrovascular disease (I60-169), and non-CVD related causes. This approach has been previously validated by the CDC and used in many CDC reports.^25^^,^^26^^,^^27^

### Quantification and statistical analysis

Software statistical package R, version 4.2.3, (R Project for Statistical Computing, http://www.r-project.org) was used to perform all statistical analyses, with *p* < 0.05 on both sides indicating statistical significance.

Participant demographic characteristics are presented in the form of descriptive statistics. The mean ± standard deviation of continuous variables was tested using an independent sample t-test, and the numbers and percentages of all categories of variables were evaluated using the χ^2^ test to compare the differences between the IM and UC groups. The proportionality of hazards assumption was assessed using the Schoenfeld residuals technique^28^ (Figure S6), with the proportional hazard assumption being satisfied (*p* = 0 · 44 for testing departures from proportionality), and Cox proportional hazards regression models were used to estimate the association between integration management and CVD-specific and all-cause mortality. An initial multivariable Cox regression model was performed, including demographic factors, clinical factors, laboratory test results, and medications. In addition, to account for the inherent limitations of observational studies (non-randomized treatment administration), we used propensity score methods to reduce the effects of confounding factors. Individual propensities for the receipt of IM were estimated using a multivariable logistic regression model that included the same covariates as the Cox regression model. Associations between IM and CVD-specific or all-cause mortality were then estimated using multivariable Cox regression models and three propensity score methods.

Inverse probability weighting (IPTW) was used for the primary analysis. In this analysis, the predicted probabilities from the propensity score model were used to evaluate the stabilized inverse probability weights.^29^ Kaplan–Meier curves and Cox models that used the inverse probability weights were reported. The nonparametric bootstrap method was used to obtain 95% pointwise CIs for the IPTW Kaplan–Meier curves. We conducted a secondary analysis using propensity score matching and another analysis that included the propensity score as an additional covariate. In the propensity score matching analysis, the nearest-neighbor method was applied to create a matched control sample. Orientation matching was conducted in a 1:1 ratio using both the IM model and the routine care model (The routine care groups comprised preexisting primary medical services, with the exception of standardized training on hypertension and antihypertensive treatment for township medical staff), with a caliper value of 0.1. The impact of the IM and conventional care models on CVD-specific and all-cause mortality was assessed using both unadjusted and adjusted Cox proportional hazard models. A multivariate Cox proportional hazards regression model was applied after inverse probability weighting and propensity score matching to examine the impact of the two groups on all-cause and CVD-specific mortality. The Kaplan-Meier method was used to plot the cumulative risks for all three datasets.

Additional sensitivity analyses included the same set of analyses to test the robustness of our findings: (1) using multiple imputation to handle all missing covariates to assess the impact of missing variables on our results; (2) randomly selecting data from 10 out of 17 townships for the same analysis for internal validation; (3) using the Fine-Gray competing-risk regression to estimate the subdistribution HR (SHR), considering non-CVD-related mortality as a competing event, the cumulative probability of recurrence was estimated using the CIF, and plot stacked CIF curve; and (4) excluding death cases that occurred within the first six months of follow-up to decrease reverse causality bias. Multiple imputation was used to handle missing data, and model estimates and standard errors were calculated using Rubin’s rules.^30^
