# Supplementary material for: Effectiveness of integrated management on hypertension and mortality in rural China: A CHHRS study
Source: iScience. 2024 Aug 31;27(10):110865. doi: 10.1016/j.isci.2024.110865 (PMC11417325; doi:10.1016/j.isci.2024.110865)
Supplement: Document S1. Figures S1–S7 and Tables S1–S4 [file mmc1.pdf]

iScience, Volume *27*

Supplemental information

Effectiveness of integrated management on hypertension and mortality in rural China: A CHHRS study

Chao Yu, Yumeng Shi, Peixu Zhao, Tao Wang, Lingjuan Zhu, Wei Zhou, Huihui Bao, and Xiaoshu Cheng

Supplemental information

Document S1

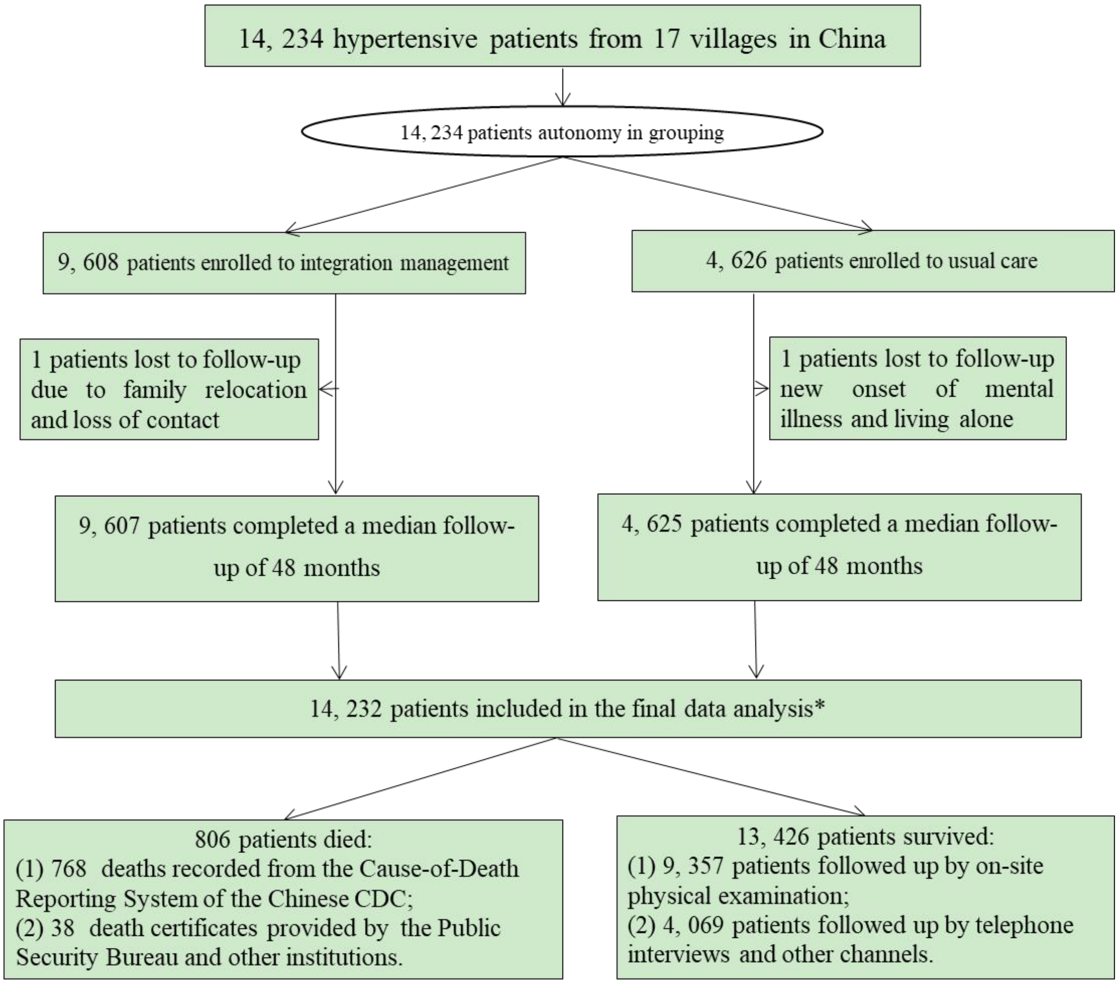

Figure S1. Flow chart of the study participants, related to STAR Methods

*The high rate of follow-up in this study (as of August 2022) can be attributed to the recent establishment of a complete COVID-19 virus nucleic acid and Vaccine Information Registration System in China. As a result, the accuracy and completeness of the resident information registration rate reached almost 100%, making it easier to track and monitor participants throughout the study.

Abbreviations: *CDC*, Centers for Disease Control and Prevention.

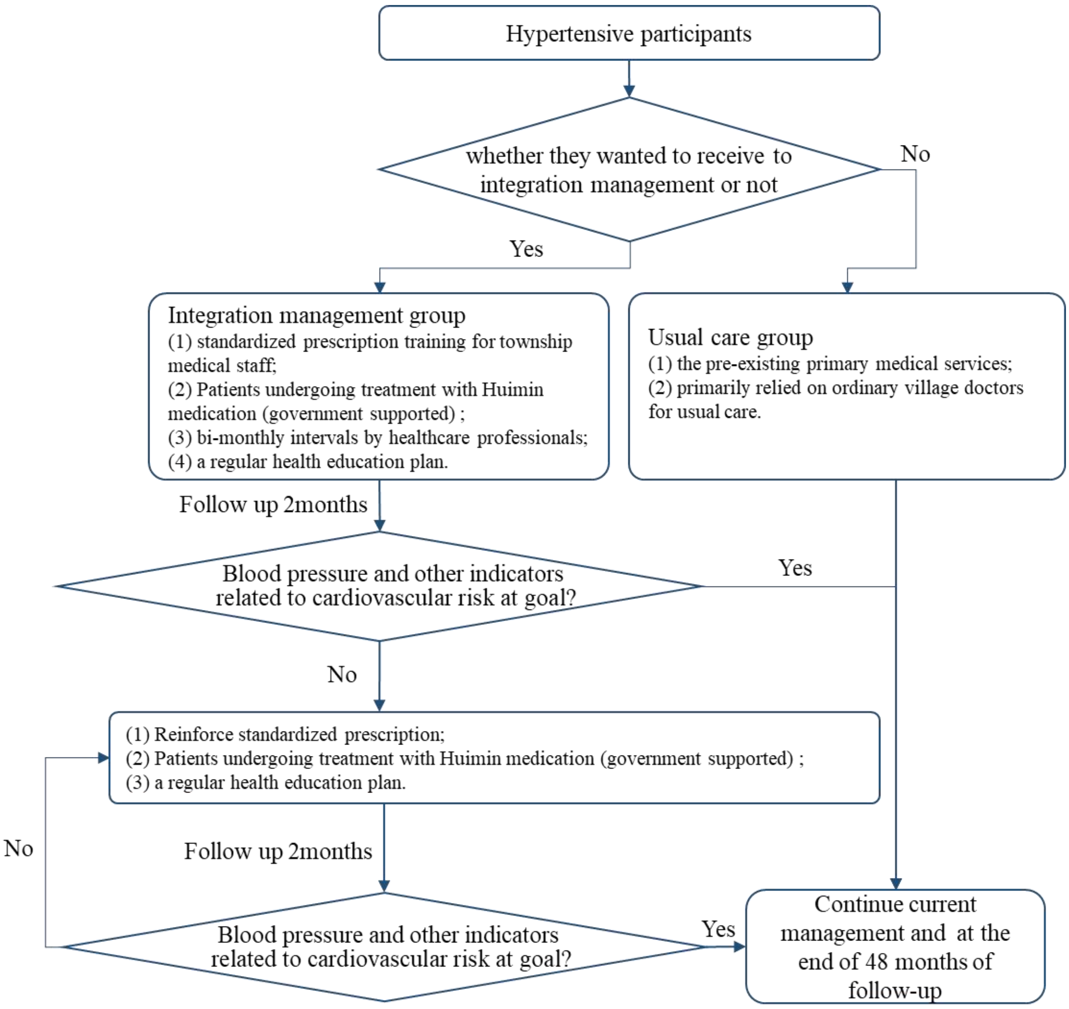

Figure S2. Management protocol of the study , related to STAR Methods

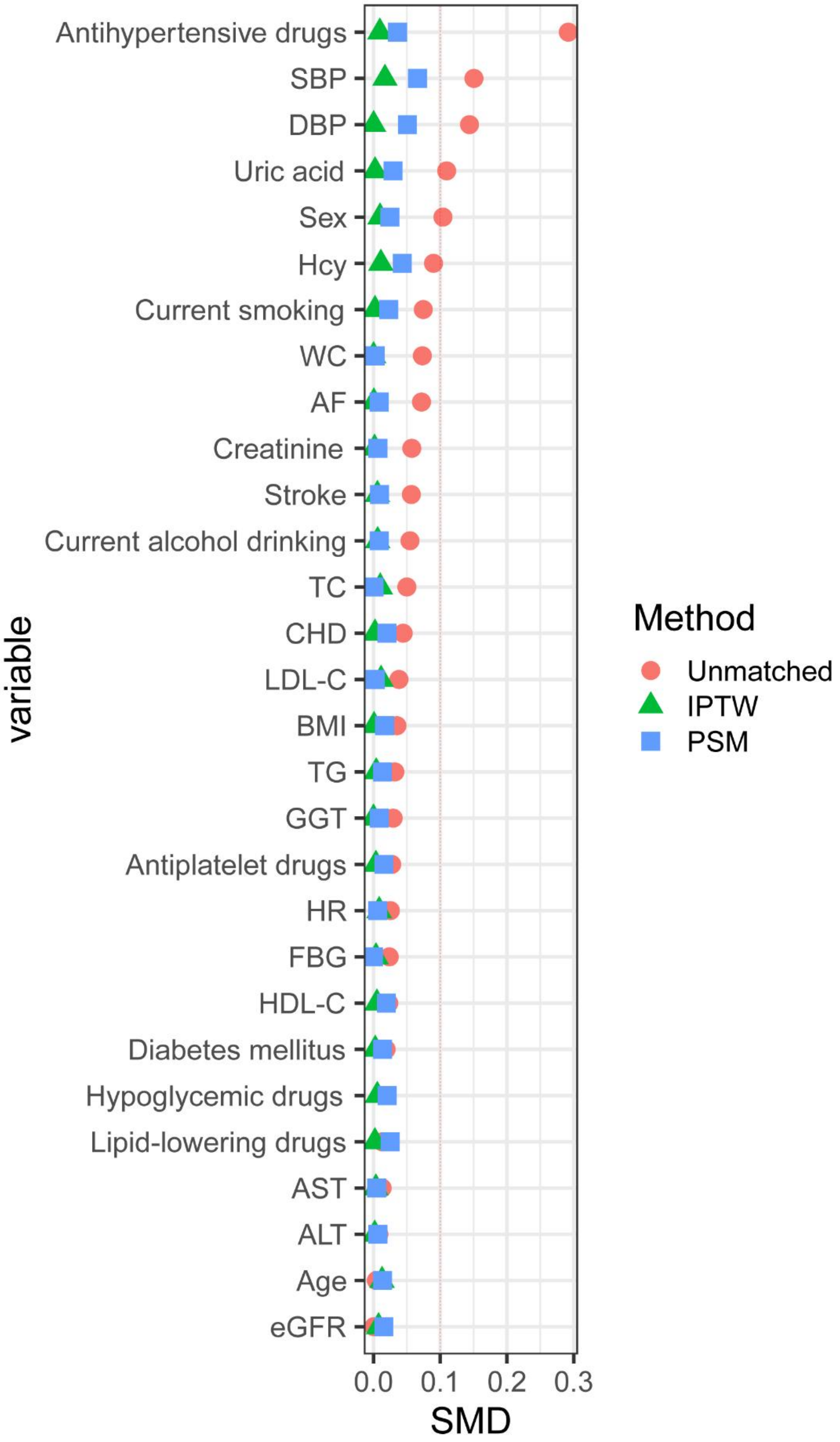

Figure S3. Standardized mean differences in the unmatched, IPTW-matched and PSM-matched sampley, related to STAR Methods

Abbreviations: SMD, Standardized mean differences; *IPTW,* Inverse Probability of Treatment Weighting; *PSM*, Propensity Score Matching

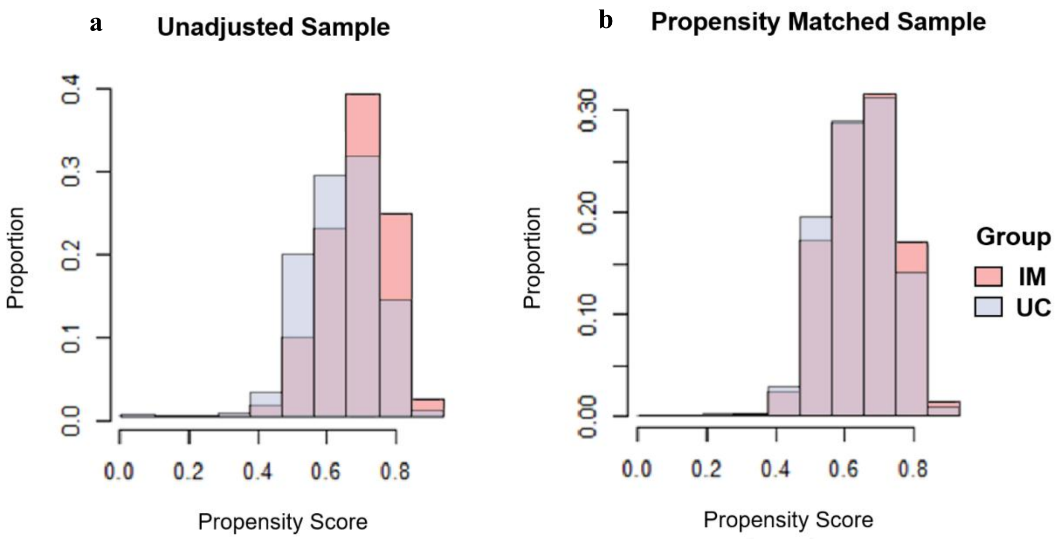

Figure S4. Distribution of the estimated propensity score for receiving integration management, among patients who did and did not actually receive the treatment. a.) Unadjusted Sample. b.) Propensity Matched Sample, related to STAR Methods

On the left, histograms of propensity scores for the unadjusted populations who were treated with integration management (IM) and were treated with usual care (UC). On the right, histograms of the propensity matched samples. Generated using the first imputed dataset. The other imputed datasets are similar and thus omitted.

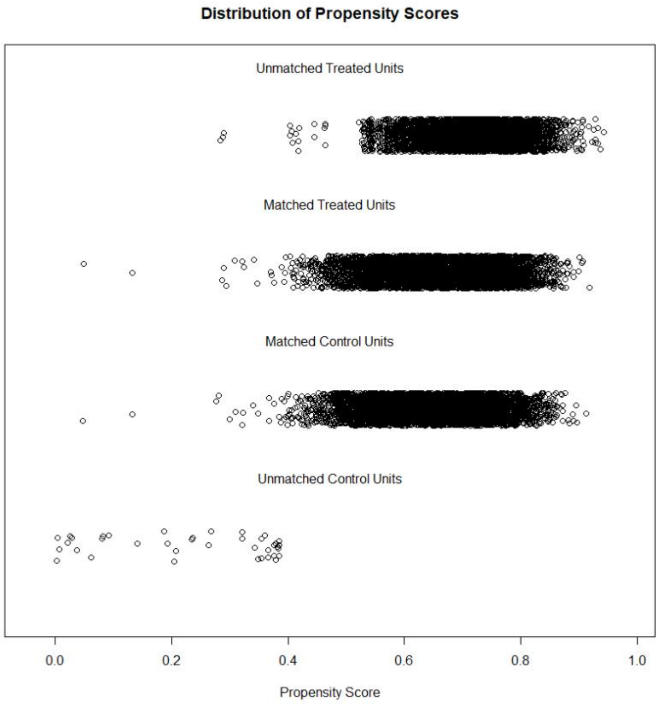

Figure S5. Scatter distribution of the estimated propensity score for receiving integration management, among patients who did and did not actually receive the treatment, related to STAR Methods

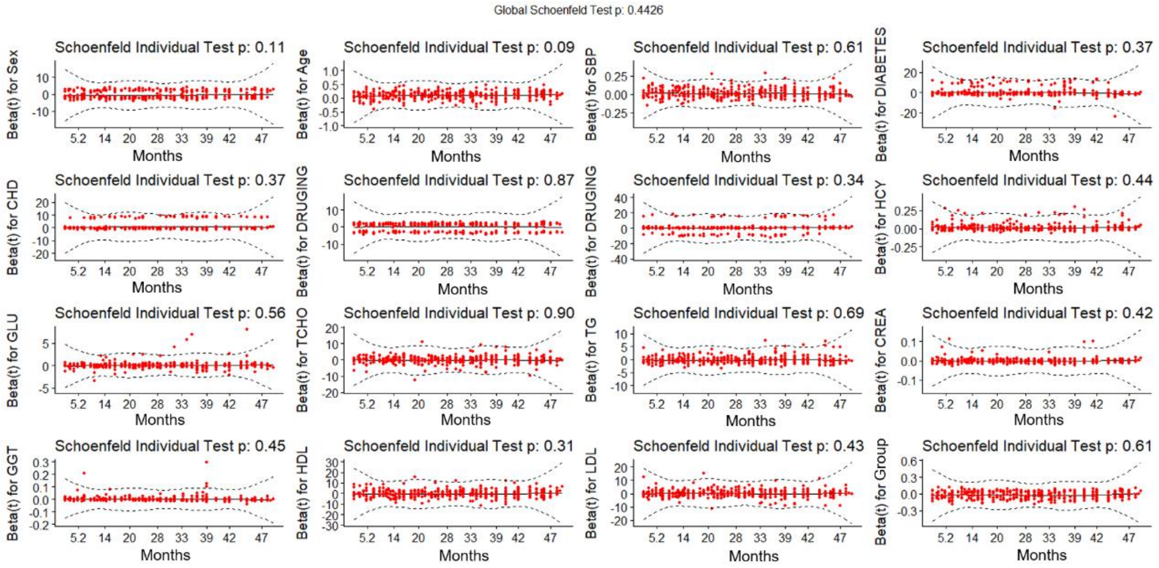

Figure S6. Analysis of the residuals of schoenfeld residuals to assess the proportional hazards assumption, related to STAR Methods

Figures represent plots of beta-coefficient estimates (log hazard ratios) for all variables included as covariates in the Cox multivariable model against follow-up (time) in months. The black solid line represents a smoothed curve of scaled Schoenfeld residuals with 95% confidence intervals (black dotted lines).

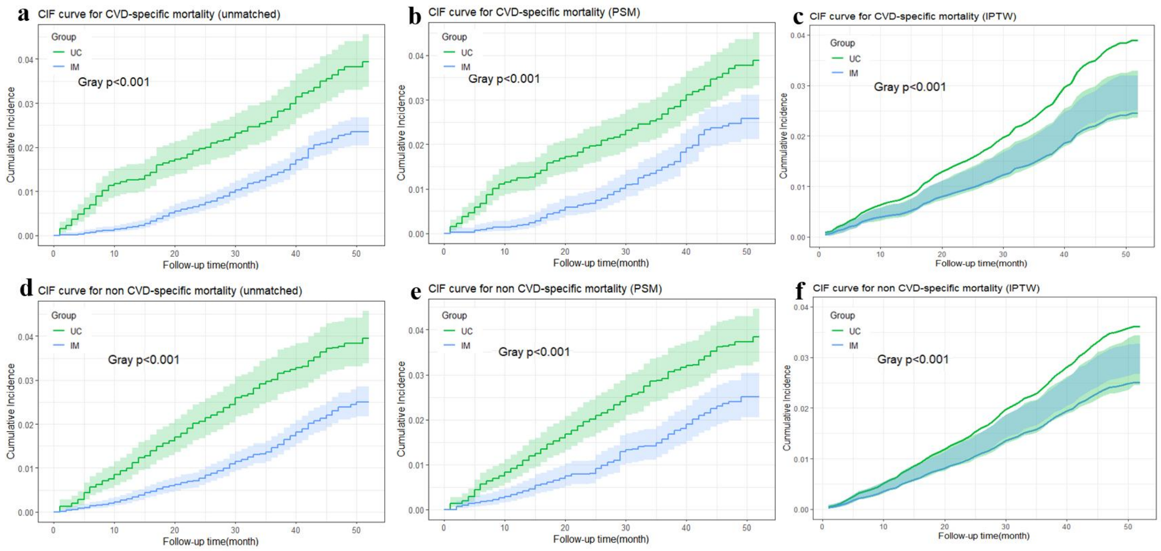

Figure S7. Cumulative incidence adjusted CIF curves based on the Fine-Gray model for the competing risk settings of CVD-Specific and non CVD-Specific Death, before after Propensity-Score Matching a.) CIF curves for CVD-Specific mortailty (unmatched). b.) CIF curves for CVD-Specific mortailty (IPTW). c). CIF curves for CVD-Specific mortailty (PSM). d.) CIF curves for all-cause mortailty(unmatched). e.) CIF curves for all-cause mortailty (IPTW). f). CIF curves for all-cause mortailty (PSM), related to Figure 1

The shaded areas represent pointwise 95% confidence intervals. 95% confidence interval of CIF after IPTW matching is calculated by bootstrap.

Table S1. Specific prescriptions for integrated management solutions, related to STAR Methods

Specific prescriptions	The antihypertensive effect of follow-up		
	Good effect	Poor effect	Still poor effect
Prescription 1	ACEI+ Folic Acid tablets	ACEI+ folate tablets +CCB	ACEI+ folate tablets +CCB+ Diuretics
Prescription 2 (Prescription 1 intolerance)	CCB+ Folic Acid tablets	CCB+ folate tablets + ARB	CCB+ folate tablets + ARB + Diuretics

Abbreviation: *ACEI*, Angiotensin-Converting Enzyme Inhibitors; *CCB*, calcium channel blocker; *ARB*, Angiotensin II Receptor Blocker.

Table S2. Sociodemographic characteristics of Patients Receiving Integration management or Usual care, related to Table 1

Characteristic	Usual care	Integration management	SMD	*P*-value
Participants	4625	9607		
Education, n(%)			0.030	0.328
Illiteracy	1416 (37.8)	2989 (38.4)		
Primary	1550 (41.4)	3256 (41.9)		
Secondary and above	782 (20.9)	1530 (19.7)		
Marital status, n(%)			0.015	0.448
In marriage	761 (20.3)	1532 (19.7)		
Unmarried, divorced / widowed	2986 (79.7)	6242 (80.3)		

Table S3. Sensitivity analyses showing hazard ratio for primary outcome using different baselines and data fitting based on interpolation algorithm, related to Table2

Methods	HR (95%CI), P value			
	Multiple imputation data[b]	Consider competing-risk[c]	Internal validation data[d]	Excluding death cases<6 m[e]
**CVD-specific mortality**				
Multivariable analysis[f]	0.58 (0.47, 0.71)<br><0.0001	0.62 (0.61, 0.63)<br><0.0001	0.71 (0.56, 0.90)<br>0.0053	0.72 (0.58, 0.89)<br>0.0031
Propensity-score analyses				
With IPTW[g]	0.53 (0.43, 0.66)<br><0.0001	0.52 (0.32, 0.72)<br><0.0001	0.73 (0.57, 0.92)<br><0.0001	0.64 (0.51, 0.80)<br><0.0001
With matching[h]	0.58 (0.46, 0.75)<br><0.0001	0.59 (0.46, 0.75)<br><0.0001	0.67 (0.50, 0.89)<br>0.0053	0.69 (0.54, 0.90)<br>0.0052
Adjusted for propensity score[i]	0.57 (0.45, 0.73)<br><0.0001	0.60 (0.46, 0.76)<br><0.0001	0.70 (0.52, 0.93)<br>0.0150	0.69 (0.53, 0.90)<br>0.0055
**All-cause mortality**				
Multivariable analysis	0.62 (0.53, 0.71)<br><0.0001	/	0.68 (0.58, 0.81)<br><0.0001	0.71 (0.61, 0.83)<br><0.0001
Propensity-score analyses				
With IPTW	0.59 (0.51, 0.68)<br><0.0001	/	0.73 (0.62, 0.86)<br><0.0001	0.66 (0.57, 0.786)<br><0.0001
With matching	0.60 (0.50, 0.71)<br><0.0001	/	0.63 (0.52, 0.77)<br><0.0001	0.69 (0.58, 0.82)<br><0.0001
Adjusted for propensity score	0.59 (0.50, 0.71)<br><0.0001	/	0.65 (0.53, 0.80)<br><0.0001	0.69 (0.57, 0.83)<br><0.0001

[a]Shown is the analysis using all the original data.

[b]Shown is the analysis using multiple imputed data.

[c]Shown is the analysis using the Fine-Gray competing-risk regression to estimate the subdistribution HR (SHR), considering CVD mortality and non-CVD mortality as competing events.

[d]Shown is a random selection of data from 10 townships for internal validation.

[e]Shown is after excluding participants died within the first six months of follow-up.

[f]Shown is the hazard ratio from the multivariable Cox proportional-hazards model, with additional adjustment for age, sex, BMI, WC, Current smoking, Current alcohol drinking, SBP, DBP, HR, diabetes mellitus, stroke, CHD, AF, HCY, TCHO, TG, LDL-C, HDL-C, UA, eGFR, AST, ALT, GGT, Creatinine, Hypoglycemic drugs, Lipid-lowering drugs, Antiplatelet drugs, Antihypertensive drugs, and laboratory tests on presentation. The analysis included all 14232 patients.

[g]Shown is the primary analysis with a hazard ratio from the multivariable Cox proportional-hazards model with the same covariates with inverse probability weighting according to the propensity score. The analysis included 28504 patients (14232 who received Integration management and 14272 who did not).

[h]Shown is the hazard ratio from a multivariable Cox proportional-hazards model with the same covariates with matching according to the propensity score. The analysis included 9172 patients (4586 who received Integration management and 4586 who did not).

[i]Shown is the hazard ratio from a multivariable Cox proportional-hazards model with the same strata and covariates, with additional adjustment for the propensity score. The analysis included 9172 patients (4586 who

received Integration management and 4586 who did not).

Abbreviations: HR, hazard ratio; *CI,* confidence interval; *IPTW,* Inverse Probability of Treatment Weighting.

Table S4. Hazard ratios (95% CIs) for the composite endpoint for all variables included as covariates in the Cox multivariable model with inverse probability weighting by the propensity score*, related to Table 2.

Characteristic	CVD-specific mortality		All-cause mortality	
	H*R* (95%CI)	*P* value	H*R* (95%CI)	*P* value
Females	0.62(0.46,0.84)	0.0018	0.57(0.46,0.70)	<0.0001
Age,year	1.08(1.06,1.11)	0.0000	1.07(1.05,1.08)	<0.0001
BMI,kg/m^2	0.93(0.87,0.99)	0.0249	0.90(0.86,0.94)	<0.0001
WC, cm	1.01(0.99,1.03)	0.5034	1.01(1.01,1.02)	0.0976
Current smoking	1.20(0.92,1.58)	0.1844	1.02(0.84,1.23)	0.8714
Current alcohol drinking	0.92(0.67,1.26)	0.6064	0.83(0.66,1.04)	0.1059
SBP, mmHg	1.02(1.01,1.03)	<0.0001	1.01(1.01,1.02)	<0.0001
DBP, mmHg	0.99(0.98,1.01)	0.5764	0.99(0.98,1.01)	0.1608
Heart rate, beats/min	1.01(1.01,1.02)	0.0005	1.01(1.01,1.02)	<0.0001
**Past diagnoses**				
Diabetes mellitus	0.66(0.42,1.04)	0.0733	0.93(0.68,1.26)	0.6327
Stroke	2.20(1.55,3.13)	<0.0001	1.77(1.36,2.30)	<0.0001
CHD	1.42(0.97,2.06)	0.0682	1.39(1.06,1.82)	0.0168
AF	2.13(1.44,3.15)	0.0002	1.65(1.22,2.24)	0.0013
**Medications at baselines**				
Antihypertensive drugs	1.21(0.93,1.57)	0.1583	1.17(0.98,1.41)	0.0912
Hypoglycemic drugs	1.58(0.89,2.82)	0.1176	1.39(0.96,2.03)	0.0852
Lipid-lowering drugs	0.78(0.40,1.52)	0.4694	0.71(0.43,1.18)	0.1917
Antiplatelet drugs	2.29(1.42,3.69)	0.0007	1.64(1.13,2.39)	0.0090
**Initial laboratory tests**				
Hcy,μmol/L	1.02(1.01,1.02)	<0.0001	1.01(1.01,1.02)	0.0001
FBG, mmol/L	1.12(1.02,1.23)	0.0215	1.10(1.04,1.16)	0.0013
TC, mmol/L	0.85(0.64,1.11)	0.2329	0.97(0.79,1.18)	0.7492
TG, mmol/L	0.80(0.68,0.94)	0.0056	0.81(0.72,0.91)	0.0005
AST, mmol/L	1.01(1.00,1.02)	0.0131	1.01(1.00,1.02)	0.0177
ALT, mmol/L	0.98(0.96,1.00)	0.0830	0.98(0.97,0.99)	0.0084
Uric acid, mmol/L	1.01(1.01,1.02)	0.0029	1.01(0.99,1.01)	0.1690
Creatinine, μmol/L	0.99(0.99,1.01)	0.6125	1.00(0.99,1.01)	0.1663
GGT, U/L	0.99(0.99,1.01)	0.5792	1.01(0.99,1.01)	0.0673
HDL-C, mmol/L	0.68(0.44,1.06)	0.0916	0.85(0.63,1.15)	0.2951
LDL-C, mmol/L	1.40(0.97,2.04)	0.0749	1.07(0.83,1.38)	0.6106
eGFR, mL/min/1.73 m^2	0.99(0.98,0.99)	0.0327	0.99(0.98,0.99)	0.0004

* This Cox multivariable model, which was inverse probability weighted by the propensity score, was designed to control for potential confounding-by-indication of the exposure (receiving integration management) and composite outcome.

It was additional stratified on sex, chronic lung disease and BMI, for which parameter estimates are not generated or shown. The results in this table are provided for the reader's information but should be not be interpreted to provide information on predictors or causes of the composite outcome
